# GAIT PARAMETERS IN OLDER BREAST CANCER SURVIVORS WITH PERSISTENT CHEMOTHERAPY-INDUCED PERIPHERAL NEUROPATHY

**DOI:** 10.1093/geroni/igad104.1206

**Published:** 2023-12-21

**Authors:** Constance Visovsky

**Affiliations:** University of South Florida-College of Nursing, Tampa, Florida, United States

## Abstract

Chemotherapy-induced peripheral neuropathy (CIPN) results from chemotherapy for breast cancer treatment. CIPN symptoms persist causing significant loss of functional abilities and increased risk of falls. This study aimed to assess the spatiotemporal gait parameters in breast cancer survivors with CIPIN using an IMU system. We hypothesized that spatiotemporal gait parameters will differ between patients with persistent CIPN as compared to normative values. Thirty-two women who completed taxane-based chemotherapy for breast cancer and who have symptomatic neuropathy >6 months following completion of chemotherapy. Gait was assessed using seven Inertial Measurement Unit (IMU) sensors positioned at the sacrum, upper and lower leg, and foot. Participants completed a 2-minute walk at a comfortable walking pace. The outcome variables assessed included gait speed, single limb support, stride length, gait cycle time, stance percent, swing percent, double support percent, and cadence. Mean age 65(10) years, gait speed was 0.95(0.2) meters/second, single limb support 37.9(2.2) % of gait cycle, stride length 1.1(0.2) meters, gait cycle time 1.2(0.1) seconds, stance percent 62.1(2.2)% GC, swing percent 37.9(2.2)% GC, double support percent 23.9(4.3)% GC, and cadence 104.6(12.3) steps/min. Gait parameters assessed in this study showed an excellent test-retest reliability (r >0.86; ICC > 0.90). This is the first study to assess gait parameters in older breast cancer survivors with CIPN. Breast cancer survivors with CIPN showed reductions in all gait parameters. Reduced gait speed and stride length are critical indicators of fall risk for women with CIPN. Our study confirmed differences in spatiotemporal parameters between patients with persistent CIPN.

